# Selection of Acetic Acid Bacterial Strains and Vinegar Production From Local Maltese Food Sources

**DOI:** 10.3389/fmicb.2022.897825

**Published:** 2022-07-19

**Authors:** Joseph Mizzi, Francesca Gaggìa, Nicole Bozzi Cionci, Diana Di Gioia, Everaldo Attard

**Affiliations:** ^1^Division of Rural Sciences and Food Systems, Institute of Earth Systems, University of Malta, Msida, Malta; ^2^Department of Agricultural and Food Sciences, University of Bologna, Bologna, Italy

**Keywords:** *Acetobacteraceae*, vinegar, acetous fermentation, wood treatment, polyphenols

## Abstract

This study investigates the isolation, identification, and fermentation performance of autochthonous acetic acid bacteria (AAB) from local niche habitats on the Island of Gozo (Malta) and their further use for vinegar production, employing local raw materials. The bacteria were isolated from grapevine berries and vinegar produced in the cottage industry. Following phenotype and genotype identification, the AAB were ascribed to the genera *Acetobacter, Gluconobacter*, and *Komagataeibacter*. A mixture of selected AAB was tested as an inoculum for vinegar production in bench fermenters, under different conditions and substrates, namely, grapes, honey, figs, onions, prickly pear, and tomatoes. The bench fermenters were operated under semi-continuous fermentation where working volumes were maintained by discharging and subsequent recharging accordingly to maintain the acidity in fermenters by adding 30–50 g/l of acetic acid for optimal *Acetobacteraceae* performance. Finally, the vinegar products obtained from the different substrates were evaluated for their quality, including organoleptic properties, which showed the superior quality of wood-treated vinegar samples with respect to neat vinegar samples.

## Introduction

Vinegar production is a millennia-old process. Vinegar is an acidic liquid resulting from a two-step fermentation process, in which a microbial consortium mainly consisting of *Saccharomyces cerevisiae* and acetic acid bacteria (AAB) transforms any edible carbohydrate-rich source into a liquid food rich in acetic acid (Garcia-Parrilla et al., 2017). Beyond its food preservative action, vinegar possesses some health properties (Ho et al., 2017). The acetic acid bacteria (AAB) are known as highly versatile microorganisms of great biotechnological relevance. They are Gram-negative or Gram-variable, ellipsoidal to rod-shaped cells, and have an obligate aerobic metabolism with oxygen as the terminal electron acceptor. In the first classification of AAB, two main genera were determined as *Acetobacter* and *Gluconobacter*, but currently, more than 12 genera are recognized and included within the family *Acetobacteraceae*. These microorganisms can produce high concentrations of acetic acid from ethanol, which makes them important to the vinegar industry (Sengun and Karabiyikli, 2011). The acetic acid produced by AAB during vinegar production is responsible for its characteristic aroma; AAB strongly influence the quality of vinegar, although the final quality is a result of a combination of factors, such as the raw material, technological process, and wood contact if vinegar undergoes aging step (Tesfaye et al., 2002). Two standard systems are applied for vinegar brewing: solid-state fermentation, mostly used in Asian countries, and liquid fermentation, particularly developed in Western countries. Vinegar in Europe is mainly produced by the submerged system through an aerobic process by which the ethanol in liquids (spirits, wine, or cider) is oxidized to acetic acid by AAB, in controlled stirring conditions (Gullo et al., 2014). The most common vinegar on the EU market derives from the fermentation of white and/or red grapes and apples, but interest in other substrates is on the increase. The attention is mainly focused on surpluses of vegetables (second quality for size or shape) and waste of sugar-rich foods, which could be a potential source for vinegar production. Waste utilization in the fruit and vegetable processing industry is an important challenge for governments to address, in an attempt to sustain a natural balance in the future (Roda et al., 2017). Some successful attempts have been performed with strawberries (Ubeda et al., 2013), pepper leaves (Song et al., 2014), blueberry, pineapple (Roda et al., 2017), orange fruits (Davies et al., 2017), pomegranate (Kharchoufi et al., 2018), mango, and papaya (Bouatenin et al., 2021). Grapevine cultivation dates back to millennia in the Maltese Islands; the two main autochthonous grapevine varieties are Gellewza and Girgentina. The production of vinegar from grape wine is mainly used for the preservation of the Maltese cheeselet called “gbejna,” which is pickled for around 24 h and then coated with coarsely ground black pepper (Morales et al., 2017). Typically, vinegar is produced from Maltese grapes. Substrates other than grapes were not very popular in the past, locally. The main objective of this study was to isolate new AAB strains from local niche habitats (berries from grape vines and locally produced vinegar) to exploit the local biodiversity within AAB in order to obtain strains that can be adapted to the fermentation of local substrates. The selected strains were utilized to develop an efficient reliable two-stage fermentation process to produce vinegar from locally available raw fruit and vegetable materials (e.g., figs, prickly pears, onions, tomatoes, and honey, apart from grapes). The island of Gozo, in fact, is rich in agri-food wastes; overproduction, damaged vegetables, and honey not suitable for human consumption can be recovered and used as substrates, limiting the amounts of waste.

## Materials and Methods

### Grapes and Vinegar Samples

Mature grape berries and vinegar samples from the cottage industry were collected throughout the Island of Gozo (Malta) in sterile stomacher bags and 500 ml sterile jars, respectively. The grape samples were collected over two seasons, summer 2013 and summer 2014, while the vinegar samples were collected between these two harvests. Sampling sites and the number of samples are described in Table 1. The grape berries (500 g) were collected the week before harvest. In mono-varietal vineyards, sampling was carried throughout the field following the “W” shape collection method, whereas in multi-varietal fields, a random vine was chosen as the source of sampling. In the case of isolated indigenous vines at the boundaries of the field, whole grape bunches were collected from the same vine. Random vinegar samples were collected directly from the surface of the active fermenting containers with the slow traditional static method. The fermenting vat was topped up from time to time with wine usually left over at the bottom of the barrels (the last few centimeters), as this wine may contain some sediment. These collected samples bridged the cottage production of vinegar with laboratory-produced vinegar. The collected samples were stored in insulated containers with ample oxygen by transporting them in sample bottles with large headspace (350 ml sample in 500 ml jar) and induced agitation (hanging container) to favor a rich supply of dissolved oxygen.

**Table 1:** Site of grape and vinegar sampling in the Island of Gozo, Malta.

**Site of**		**Year of**	**Grape**	**Vinegar**
**isolation**		**sampling**	**samples**	**samples**
North	Ghasri, Zebbug, Xaghra	2013–2014	/	7
East	Nadur, Qala, Ghajnsielem	2013–2014	13	4
South	Munxar, Sannat, Xewkija	2013–2014	3	3
West	Gharb, San Lawrenz, Kercem, Santa Lucija	2013–2014	13	14
Center	Rabat, Fontana	2013–2014	6	4

### AAB Isolation and Culturing

#### Isolation From Grape Berries

To ~50 g of berries, 47 ml of sterile distilled water and 3 ml of filtered absolute ethanol (99.8%; Sigma-Aldrich; Milan, Italy) were added. The suspension was mixed for 1 min, using the Seward Stomacher 400 (Seward Ltd, Technology Center, Worthing, West Sussex, UK) and incubated at room temperature for 7 days (Gullo et al., 2009). The obtained enrichment was inoculated on the surface of Glucose-Yeast-Extract-Calcium Carbonate Agar (GYC medium; glucose 5%, yeast extract 1%, CaCO3 1%, agar 1.5%, pH 6.8 ± 0.2; Scharlau Microbiology, Barcelona, Spain) supplemented with 100 mg/l of natamycin (Sigma-Aldrich, cod.: 32417) to inhibit fungal growth and incubated at 30°C for 5–10 days (Sengun and Karabiyikli, 2011). If no growth was noted, the enrichment broth was centrifuged, and inoculum from the sediment was streaked again on the GYC media plates. Where there was an overgrowth, a 1:10 dilution method up to five log reduction was applied. Tiny colonies with a clear halo were re-streaked onto WL nutrient agar (Oxoid, Ltd., Basingstoke, Hampshire, England), supplemented with cycloheximide (2 mg/l) (Sigma-Aldrich), and incubated at 25°C for 42–72 h. Colonies with a yellow halo were selected for further analysis and named with the prefix “G.”

#### Isolation From Vinegar

About 10 ml of each vinegar sample was inoculated in 30 ml sterile nutrient broth (Sigma-Aldrich, Milan, Italy) with the addition of filtered absolute ethanol and acetic acid (Sigma-Aldrich, Milan, Italy) to obtain a final concentration of 2 and 1%, respectively. The broth suspension was then incubated for 7 days at room temperature, then sub-inoculated onto AE agar medium (Entani et al., 1985) supplemented with natamycin (100 mg/l) and incubated at 30°C for 2–7 days. If no growth resulted on the sub-inoculated agar after 7 days, further AE medium plates were streaked and incubated for 7 days. This step was repeated three times at 7 days of interval before repeating the whole process on a fresh vinegar sample. When growth occurred, small colonies were re-streaked on WL nutrient agar (Scharlau Microbiology) supplemented with cycloheximide (2 mg/l) (Sigma-Aldrich) and incubated at 25°C for 48–72 h. Colonies were frozen for further analysis and named with the prefix “V.”

### Phenotypic and Genotypic Characterization of Isolated Strains

The fresh colonies were first characterized by Gram staining (Silva et al., 2006). The oxidase test was then performed using the Microbact Oxidase strips (Oxoid, Milan, Italy), and for the catalase test, colonies were placed onto a clean slide with a drop of a freshly prepared solution of 3% H_2_O_2_. Among all isolates, those being Gram-negative, oxidase-negative, and catalase-positive were purified by streak-plate technique and stored at 4°C on WL nutrient agar supplemented with cycloheximide (2 mg/l) and sub-inoculated every 6–8 weeks on the same medium. All isolates were also stored at −80°C. A single colony from an overnight culture (WL nutrient agar at 30°C) was picked up and suspended in 500 μl of sterile distilled water. The DNA extraction was carried out using the InstaGene Matrix kit (Bio-Rad, Milan, Italy) following the manufacturer's instructions. Extracted DNA was used for the amplification of the 16S rRNA gene with primers 27f (5′-GTGCCAGCAGCCGCGG-3′) and 1492r (5′-TACGGYTACCTTGTTACGACTT-3′), according to Gaggìa et al. (2013). The restriction analysis of amplified 16S rRNA (ARDRA) was performed according to Di Gioia et al. (2002). The reaction was performed by mixing 10 μl of PCR product, 17 μl of deionized water, 2 μl of 10X restriction buffer, and 1 μl of the enzyme (*Alu*I and *Hae*III; Thermos Fisher) and incubating at 37°C for 10 min. The enzymes were then inactivated by heating the reaction mixture to 65°C for 15 min. The reaction products were analyzed by agarose (2% w/v) gel electrophoresis and visualized with the gel acquisition system Gel DocTM XR (Bio-Rad). The purified PCR products of the representative strains obtained from the ARDRA analysis were delivered to Eurofins MWG Operon (Ebersberg, Germany) for sequencing. Sequence chromatograms were edited and analyzed using the software programs Finch TV version 1.4.0 (Geospiza Inc., Seattle, WA, USA). DNAMAN software (Version 6.0, Lynnon BioSoft. Inc., USA) was used to obtain consensus sequences that were processed for the species assignment through the GeneBank Database (NCBI), by using the nucleotide BLAST (Basic Local Alignment Search Tool; http://www.ncbi.nlm.nih.gov/BLAST/). The relatedness of the isolated strains within the Acetobactereaceae family was achieved with a phylogenetic tree reconstructed by sequence alignment, using the Neighbor-joining (NJ) algorithm (Saitou and Nei, 1987) and Mega version 5.0 (Tamura et al., 2011). The bootstrap values were calculated based on 1,000 replications in order to evaluate the confidence levels of the nodes.

### The Raw Substrates for Vinegar Production

For this study, the choice of the raw materials (locally grown grapes, honey, onions, figs, prickly pears, and tomatoes) was based on their availability and their potential exploitation for vinegar production. On the island of Gozo, farmers grow a large number of international and local grape varieties. However, the indigenous varieties contain a low sugar content (15–19% brix) when mature (Theuma et al., 2015), compared to locally grown international varieties (21–25/26%) (Herrera et al., 2003; Fernández-Novales et al., 2009). The international variety chosen for this study was the Primitivo variety, which occupies 12 tumoli of land utilized for grapevine production (Monte, 2013). Another typical agricultural product, unique to the Maltese Islands is honey (Attard and Mizzi, 2013). Although honey is a sought-after product (Attard and Douglas, 2017), by-products of honey extraction and honey left over in hives for extended periods are not fit for direct human consumption and may be considered as a potential starting material for vinegar production. Hence, it can be diluted to obtain a brix content of 18% solution and fermented. One of the main crops that has made it to international markets is the onion. However, high quality for export has been achieved by grading onions by appearance and size. Over-sized onions, which may result due to abundant precipitation, may be crushed and used for vinegar production. The fig tree is renowned for its delicate fruit, which tends to spoil easily and reach maturity over a short period of time. Unless preserved at cool temperatures (Owino et al., 2004), this fruit perishes quickly, hence its potential use for vinegar production. Locally, the prickly pear is primarily used as a wind breaker at the boundaries of fields. Unlike its use as a crop in most Mediterranean countries, locally the prickly pear is an underutilized crop, which may be considered as a candidate for vinegar production. The island of Gozo is also renowned for its tomato industry, which is supported by financial grants or other forms of assistance to farmers. Being one of the major cash crops, its production, at times, is very abundant and hence its potential use as a fermentable substrate.

### Fermentation Processes

**Figure 1:**
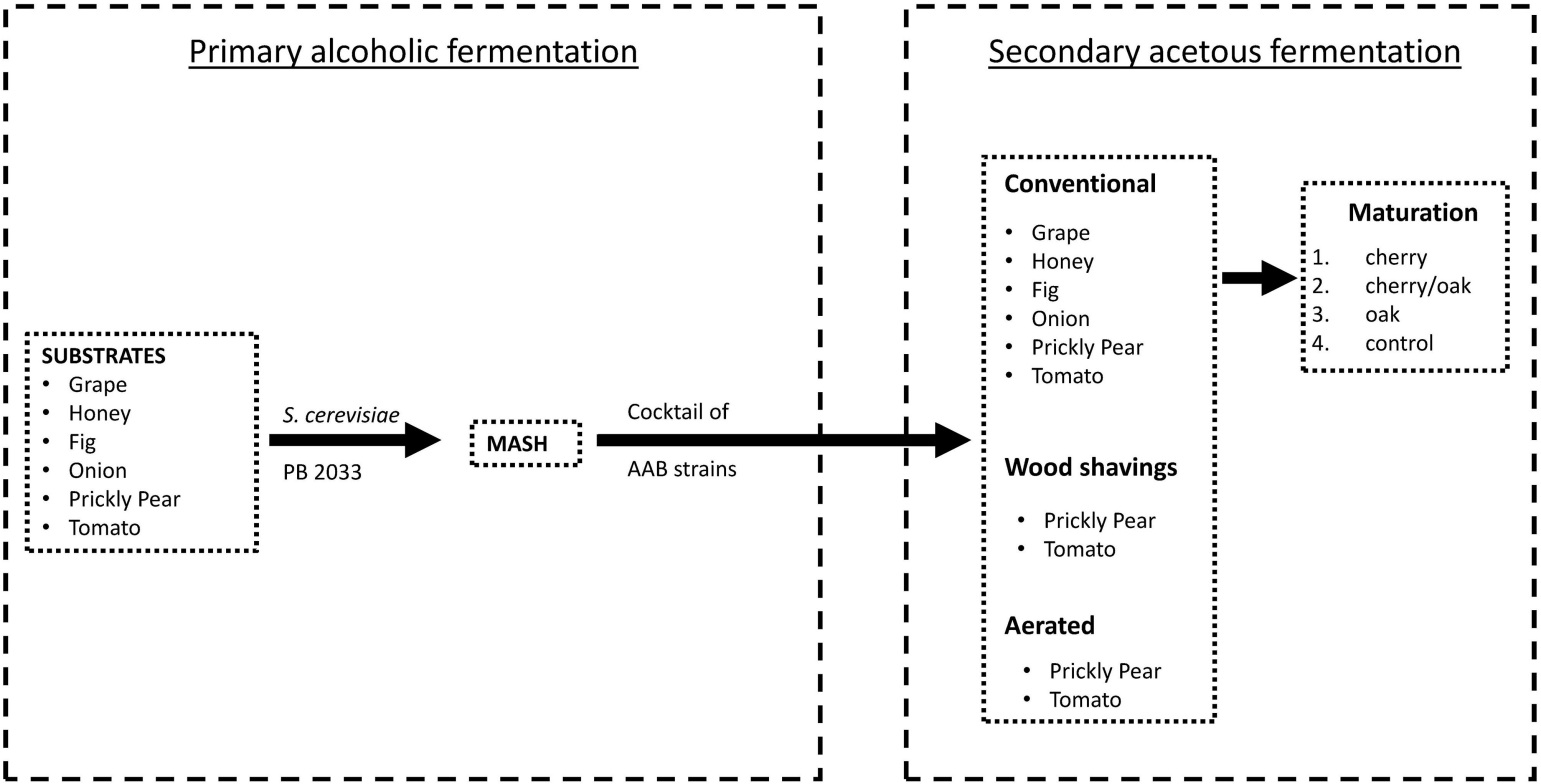
Schematic representation of both alcoholic and acetous fermentations.

The alcoholic and acetic acid fermentations proceeded separately according to the scheme shown in Figure 1. Locally grown grapes, figs, honey, onions, prickly pears, and tomatoes were chosen as substrates for the fermentation process. These raw materials were crushed, where applicable, using a traditional wine grape crusher. In order to standardize the sugar content of the substrates and obtain the desired brix value, either dilution with deionized water (as in the case of honey) or chaptalization (the addition of beetroot sugar and water) was performed. The resultant liquid ready for fermentation was denominated as wort (Grieson, 2009). The grape samples were processed immediately after collection to retain the highest level of the desired microorganisms present on the berries, without inducing any viable but non-culturable state.

#### Alcoholic Fermentation

Fifteen-liter food-grade plastic containers were used as bench fermenters. About 12 L of wort were prepared for each substrate, leaving ample headspace for any frotting or possible floating that might take place. Nutriferm Vit (Supervit, Enartis, Novara, Italy) was added to guarantee the correct nitrogen supply at 0.1% (w/v), according to the manufacturer‘s instructions. Pectolytic enzymes (Endozym Cultivar, Pascal Biotech, Brescia, Italy) were also added at 2 g/hl. *Saccharomyces cerevisiae* PB 2033 (Collection de Levures d‘Intérêt Biotechnologique, CLIB; INRA, Paris, France) was regenerated and inoculated into the substrate. The initial yeast inoculum was 2% (V/V) of a 0.5 McFarland suspension. The content of total soluble solids (TSS) of the raw material and wort was measured by using a refractometer (Brix %) (Atago 2363, Atago Co. Ltd., Japan). The fermentation progress was followed by reading the specific gravity (S.G.) using a hydrometer for rapid and easy estimation of the alcohol produced as the fermentation proceeded. When the S.G. value of about 1.020 was reached, the sugar to alcohol conversion was almost complete. At this point, this alcoholic liquid or mash was used for the acetous fermentation.

#### Acetous Fermentation

The secondary acetous fermentation was performed as described in Figure 1. The mash of grapes, honey, figs, onions, prickly pears, and tomatoes was inoculated with a consortium of selected AAB, following isolation and identification. The cocktail of AAB (*A. pasteurianus* V20, *K. saccharivorans* G1 and G10, *G. oxydans* G21, and *Gluconobacter japonicus*/*frauterii* G22) was prepared by sub-culturing the individual strains on a culture broth composed of yeast extract 1.0% (Sigma-Aldrich), K_2_HPO_4_ 0.1% (Sigma-Aldrich), MgSO_4_.7H_2_O 0.02% (Sigma-Aldrich), and ethanol 4% (Sigma-Aldrich) and incubated at room temperature for 48 h and then using an equal amount of each culture to inoculate the fermenters. The 3-L bench fermenters were filled with the mash substrate and covered with a cheese cloth to allow the fermentation, and the alcoholic degree was adjusted between 7 and 10% (Cirlini, 2008). A nutrient mixture (Acetozym DS plus2, Heinrich Frings GmbH & Co. KG, Rheinbach, Germany) was dissolved in the mash at a rate of 0.1%. Every fermentation was carried out in triplicates, having three cycles per treatment. The mash substrates of prickly pear and tomato were also subjected to two further acetous fermentation processes. (a) The use of wood shavings, where a fermenter with pre-sterilized beech wood shavings is utilized to carry out fermentation. The wood chips were placed in a pot, covered with ample water, brought to boil, and simmered for 2 h. When the wood shavings cooled to room temperature, they were added to the fermenter at a rate of 2% (w/v). (b) By aeration, where the fermenter was subjected to aeration using a regulated air pump at a constant rate of 0.25 l min^−1^. Conventional, wood shavings, and aerated fermenters were allowed to ferment simultaneously. Acetic acid bacteria were adapted to the new substrate prior to the fermenters with discharging and recharging cycles, according to the methods described by Singh and Singh (2007) and Krusong and Vichitraka (2010). Each bench fermenter was monitored for temperature changes every 30 min using Nova 5000 MultiLogPRO data loggers (Fourier Systems Ltd., Illinois, USA), while the pH and the acidity values were recorded on a daily basis by titration against 0.5 N NaOH (Sigma-Aldrich Chemie GmbH, Steinheim, Germany) and using phenolphthalein as indicator. All bioreactors were repetitively batch fermented, initiated at 3% acidity and proceeded to about 5% acidity, discharged one-third to half the volume, and replenished with more mash and nutrients to repeat the cycle.

#### Vinegar Maturation by Accelerated Aging

Four 700 mL samples of each vinegar produced from the conventional fermentation process were poured into 750 mL jars and were labeled according to the wood chips introduced: cherry, a mixture (1:1) of cherry and oak, oak, and neat (control). French oak and American cherry were used (I V Portelli & Sons Ltd, Rabat, Malta). The wood was first treated by submerging and stirring for 30 min in the respective vinegar, then drained off, and allowed to dry. The rate of application of wood chips was 2% (Tesfaye et al., 2004). After 15 days, the six vinegar samples from the conventional fermentation were transferred to new jars using a stainless steel colander to remove the wood chips and re-labeled according to the type of wood it has been matured in.

### Physicochemical Analysis

Apart from the *in situ* tests like the total soluble solids (Brix %), acidity, pH, and specific gravity, the rest of the analyses were carried out on the mature batches. These analyses included the Folin-Ciocalteu method for total polyphenols as described by Attard (2013) and the anthocyanin content using UV-Vis spectrophotometric analysis at A420/A520 (Ivanova et al., 2010; Theuma et al., 2015). The samples collected at different stages of both fermentations and after maturation in wood were stored at 4 ±1°C to be processed together. The acidification rate was calculated as described by Ndoye et al. (2007). The analysis was performed in triplicate.

### Organoleptic Evaluation

The six panelists, who volunteered for the sensory analysis, had extensive expertise in vinegar-based sauce tasting. The participants were briefed on the specific sensorial parameters, which were pungency, winy character, ethyl acetate, wood, sweet aroma, and general impression. The panelists had to taste one set of vinegar samples (control and wood matured vinegar for each of the six substrates) at a time to evaluate the attributes, according to Tesfaye et al. (2010). The participants were instructed to judge the vinegar samples, and the data obtained from the sensory analysis were expressed numerically on a 10-point hedonic scale (one being the least acceptable and 10 being the most pleasant) and tabulated accordingly. Standard solutions for these attributes were also provided as reference points, to obtain a coherent evaluation of the sensory characteristics of the attributes (Chambers and Koppel, 2013). The results were grouped into five categories with outstanding for points 9–10, standard vinegar for 7–8.99, commercial for 5–6.99, below commercial for 3–4.99, and spoiled for 1–2.99, according to Kocher et al. (2012).

### Data Collection and Analysis

The datasets generated from the fermentation monitoring analysis and batch analysis of samples were tabulated. The data collected from the questionnaire and from the organoleptic evaluation were expressed numerically and tabulated accordingly. The statistical analysis was performed by using the Statistical Package for Social Sciences (SPSS) V20 and XLSTAT version 2014.4.04 (Addinsoft). Analysis of variance (one-way ANOVA) and the Bonferroni *post-hoc* test were carried out to determine any significant differences between the mean values of the different treatments. The *p*-values obtained for correlations between the different variables were computed by the chi-square and the Friedman tests depending on the data type being analyzed. Principal component analysis with Pearson correlation statistics was carried out to determine any potential clustering of samples based on their characteristics. The level of significance was considered at *p* ≤ 0.05.

## Results

### Strain Isolation and Genotypic Identification

**Figure 2:**
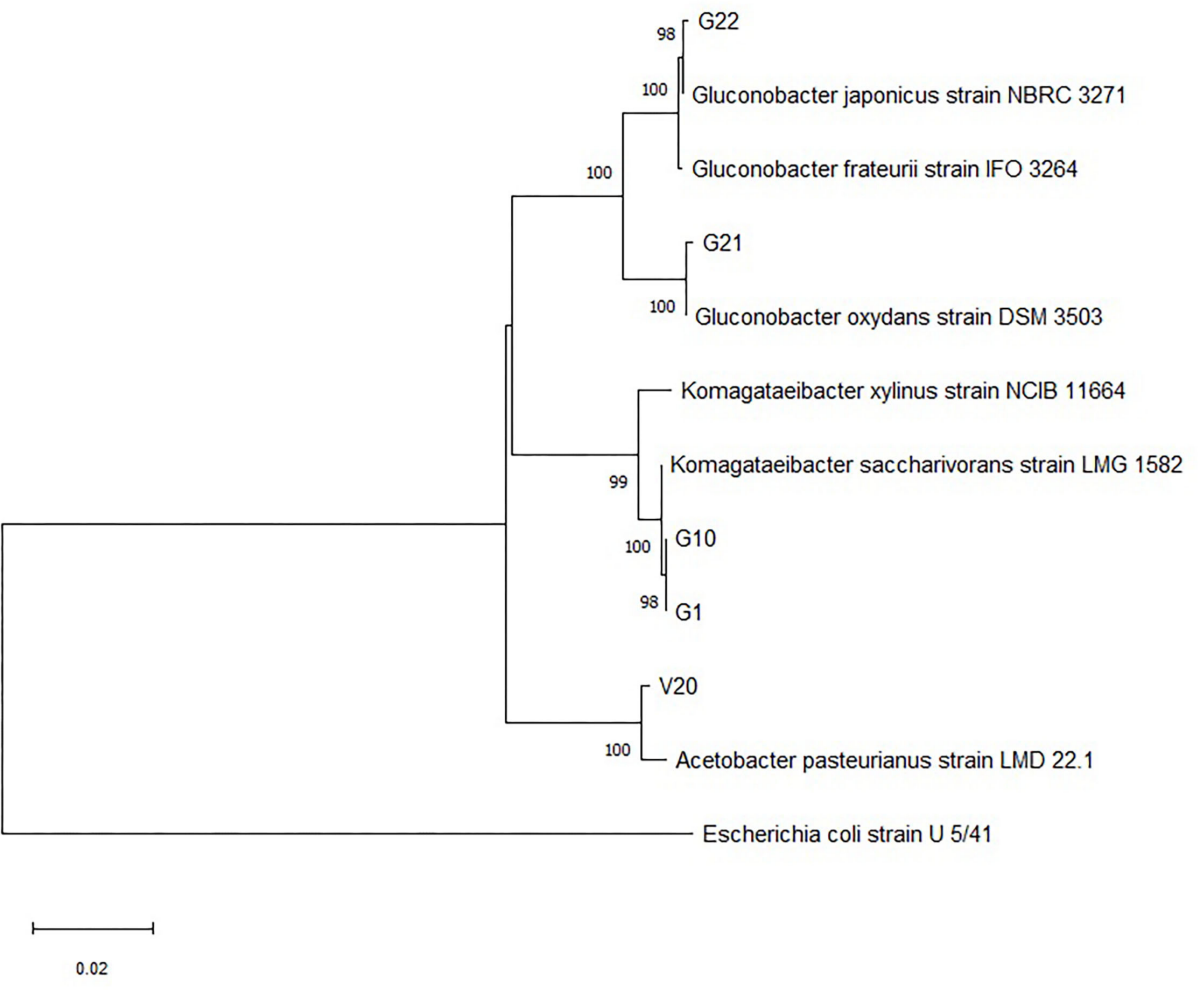
Phylogenetic tree showing the location of the selected strains based on 16S rDNA gene sequences, using the neighbor-joining (NJ) algorithm and Mega version 5.0. The tree was rooted using sequences of *Acetobacteraceae* present in the NCBI database, and *Escherichia coli* U5/41 was used as an outgroup. Bar scale indicates phylogenetic distance. The numbers at the branches represent bootstrap values (100 bootstrap re-sampling).

The colonies that were able to grow in the GYC plates and identified as Gram-negative, oxidase-negative, and catalase-positive were subcultured onto WL agar for further characterization. Overall, 32 presumptive AAB strains were isolated from grapes harvested in 2014 and grapes harvested in 2013 and vinegar samples. The fingerprinting analysis by ARDRA and the further 16S rRNA sequencing allowed for discrimination among the isolates. Supplementary Figures 1, 2 show electrophoretic gels of the restriction profiles of the 30 strains (two of the samples failed to grow when sub-inoculated) with *Alu*I and *Hae*III enzymes. The fingerprinting analysis evidenced that restriction enzymes produced three groups for *Alu*I and four groups for *Hae*III (Supplementary Table 1), with five representative strains to be sequenced (G2 restriction analysis with *Hae*III was repeated and the profile fell within cluster three). The sequencing led to species identification as presented in Table 2. All the bacteria were confirmed to belong to *Acetobacteraceae*, in particular to *Komagateibacter* spp. (strains G1 and G10), *Gluconobacter* spp. (strains G21 and G22), and *Acetobacter* spp. (strain V20). Species assignment was unique except for some strains. The sequences were then used to construct the phylogenetic tree (Figure 2) that clearly shows the different grouping of the samples and their relatedness within the *Acetobacteraceae* family. Most of the isolates (cluster 1) correspond to *Komagataeibacter* spp. In particular, all the investigated strains were found to belong to *Komagataeibacter saccharivorans*. Clusters 2 and 3 correspond to *Gluconobacter oxydans* and *Gluconobacter japonicus/frateurii*, respectively. The strain V20, whose profile with *Alu*I differs from the common pattern identified with *Hae*III, is 100% closely related to *Acetobacter pasteurianus*. These are all species commonly associated with vinegar production (Luzón-Quintana et al., 2021). *Komagataeibacter* spp., prior to the re-classification (Euzéby, 2013), formed part of the *Gluconacetobacter* and *Acetobacter* genera (Yamada et al., 2012) that were described as the major acetic acid producers in industrial vinegar fermenters with a propensity to tolerate relatively high (>6%) acetic acid concentrations (Schüller et al., 2000; Matsutani et al., 2011; Slapšak et al., 2013). Moreover, *K. saccharivorans*, together with *K. xylinus*, is reported as a major bacterial cellulose-producing species (La China et al., 2021). The isolates came from different samples of grape and vinegar. The grape samples were of a foreign origin, relatively recently imported vines, and isolations came from white table grapes and red wine grapes. Some samples were of an unknown local variety, one red table grape variety, and the other white wine/table grape variety, respectively. Their location varied from west to east of the island, showing that these *Acetobactereaceae* species are well-established and ubiquitous. The isolates from the vinegar samples came from the village of Gharb on the west side of the island except for V21 whichoriginated from Munxar. The cluster associated with *G. oxidans* comprises strains from both healthy and spoiled grapes; *G. oxidans* G11 was sampled together with G12 and V2 (*K. saccharivorans*), and they all were sourced from the same farmer who has been cultivating his own grapes and producing vinegar for decades. There is a variation in species that demonstrates how the environmental conditions dictate the type of indigenous microbiota by which the local vinegar products are produced, in accordance with Solieri and Giudici (2009); Sengun (2015). *Gluconobacter* spp. show a weak resistance to acetic acid (Prust et al., 2005; Matsutani et al., 2011). The dehydrogenase enzyme in the cell membrane facilitates its survival in nutrient-rich environments and can metabolize sugars (Moonmangmee et al., 2000; Adachi et al., 2003) and sugar acids, which other organisms cannot assimilate. It can be also found in beverages like wines and beers, and also in soft drinks, causing spoilage and off-flavors (Prust et al., 2005). The last cluster is ascribed to *Gluconobacter japonicus*/*frauterii*; the identification score did not allow a clear assignment to a species. *G. japonicus* is able to metabolize a wide range of saccharides for the production of acid. Fructose, sorbitol, glycerol, sucrose, and ethanol are weakly metabolized into acid (Malimas et al., 2009). *G. frateurii* produces acids from saccharides including sucrose but not from fructose, lactose, dulcitol, and arabinose (Kommanee et al., 2012). This group of isolates came both from the western part and from the eastern side of the island. In this instance, grapes were of the white local varieties. *Acetobacter pasteurianus* V20 was isolated from a red wine vinegar sample collected from Gharb. Strains of *Acetobacter pasteurianus* species is a highly adaptable AAB (Azuma et al., 2009) and is a major protagonist in traditional vinegar productions with acetic acid concentrations up to 6% (Ilabaca et al., 2008; Matsutani et al., 2011; Vegas et al., 2013). Gullo et al. (2009) in their investigation of species shift during the acetification process, concluded that *A. pasteurianus* can tolerate high concentrations of alcohol and thus is more adapted to the startup of the acetous fermentation. Two less sought attributes for vinegar production for this organism are its ability to over-oxidize the acetic acid to carbon dioxide and water (Matsutani et al., 2011), and to synthesize bacterial cellulose (Azuma et al., 2009). A mixture of the strains *A. pasteurianus* V20, *K. saccharivorans* G1 and G10, *G. oxydans* G21, and *Gluconobacter japonicus*/*frauterii* G22 was used, considering the change of acetic acid concentration during the fermentation process. The inoculation of the mash with a co-culture of the selected strains is often performed in vinegar production, considering that the fermentation is carried on by a succession of strains and species, depending on the concentration of acetic acid (Vegas et al., 2010). At low concentrations of acetic acid, species of the genus *Acetobacter* predominate. When acetic acid concentrations exceed 5%, the species belonging to the *Komagataeibacter* or *Gluconacetobacter* genera predominate (Mas et al., 2014). Their individual fate and persistence, as well as their individual features, were not investigated in this study; the fermentation was simply stopped at the desired acidity level.

**Table 2:** Best-match identification phylotypes of purified PCR products deriving from the 16S rRNA amplification of the isolated strains.

**Strain ID**	**Location**	**Identification**	**Similarity (%)**	Accession Number*
G1	Rabat	*Komagataeibacter saccharivorans*	99	OM984645
G10	Sannat	*Komagataeibacter saccharivorans*	100	OM984646
G21	Kercem	*Gluconobacter oxydans*	99.8	OM984648
G22	Qala	*Gluconobacter frateurii, japonicus*	99	OM984649
V20	Gharb	*Acetobacter pasteurianus*	100	OM984647

* *Provided by GeneBank (https://www.ncbi.nlm.nih.gov/genbank/)*.

### Fermentation Process

#### Alcoholic and Acetous Fermentation

The primary alcoholic fermentation of grape, fig, honey, onion, tomato, and prickly pear was performed with the chosen yeast culture, and the conversion of the sugars into alcohol lasted for a few days. There were no distinctive characteristics for the different fermentations that progressed quite uniformly for all the substrates. The TSS content of the raw substrates and the wort, and the specific gravity readings of the wort and the mash are listed in Table 3. The acetous fermentation process for the different mash treatments is shown in Table 4. The acidity and pH values were monitored for three cycles as the fermentation proceeded (Supplementary Figures 3–5). Honey and onion matrices required ~9.6 days for complete fermentation, whereas grape, prickly pear, and tomato wines required 3–5 days. The honey, onions, and grapes were less acidic than the other two substrates, and this resulted in the final acidity obtained at the end of the fermentation process. Considering pH, there were no significant differences between the initial and final pH values obtained for all substrates (Table 4). This suggests that pH does not provide a good indication of the fermentation progress. Due to the weak acidic nature of most organic acids, this can only be determined by considering the total acidity. In fact, the percentage acidity at the beginning and the end of the fermentation process is highly significant for all the substrates (*p* ≤ 0.0001). The acetification rate varied for the different substrates (Table 4). All substrates exhibited different fermentation kinetics rates in terms of rate of acidity conversion and days of fermentation. In spite of the variation between the fermentation kinetics of the different substrates, intra-fermentation kinetics was relatively similar with a very small standard deviation for each individual substrate and treatment. The onions had the slowest conversion rate of 0.18 ± 0.02% day^−1^ with a long fermentation cycle of 9.67 ± 0.88 days, whereas the fastest rate was observed in the aerated tomato being 0.64 ± 0.13% day^−1^ with a very short fermentation cycle of 3.00 ± 0.00 days. The mean rate was 0.42 ± 0.15% day^−1^ (Table 4). The initial acidity of 3% and the final acidity of 5% were chosen to maximize the performance of acetic acid bacteria, as also recommended by Gullo et al. (2014). The fermentation results showed a consistent reproducibility independent of the substrate used. Another noticeable trend is that with each consecutive fermentation cycle, a relatively shorter time than the previous cycle was recorded, indicating an increase in the acetification rate, as the acetic acid bacteria strains were able to adapt and acclimatize to the priming of the fermenter with a fresh mash sample (Supplementary Figures 3–5). The successively shorter lag phase is concordant with De Ory et al. (2004), who performed the fermentations using different immobilization techniques. Correlations between pH and acidity were computed using Pearson correlation, showing a degree of inverse linear dependence between the two variables (Supplementary Table 2). The only exception was the prickly pear wood-treated acetous fermentation. This can be explained by the potential heterogeneous fermentation with the wood chips.

**Table 3:** List of the raw substrates used in the primary alcoholic fermentation and pre-treatment; TSS % and S.G. readings of the wort and mash.

**Raw substrate**	*TSS %	**Pre-treatment**	**TSS % wort**	*S.G. wort	**S.G. mash**
Grape	24	Crushed fruits	24	1,090	1,015
Fig	12	Crushed fruits and added sugar	18	1,055	1,010
Honey	75	Dilution to 18% brix by deionized water	16	1,060	1,010
Onion	12	Peeled, sliced and adjusted brix to 18% by addition of sugar	18	1,065	1,015
Tomato	5.5	Crushed and adjusted brix to 18% by addition of sugar	18	1,075	1,000
Prickly pear	12	Peeled, crushed and adjusted brix to 18% by addition of sugar	19	1,080	1,010

*TSS %, total soluble solid percentage; *S.G., specific gravity.

**Table 4:** Days of fermentation and acidic parameters (initial/final % acidity, rate of acidity, and pH) along the acetous fermentation; values are mean ± standard deviation.

	**Days of fermentation**	**Initial acidity**	**Final acidity**	**Rate of acidity (% day^**−1**^)**	**Initial pH**	**Final pH**
Figs	3.00 ± 0.00	2.59 ± 0.60	4.93 ± 0.13*	1.66 ± 0.03	3.14 ± 0.05	2.86 ± 0.06^ns^
Honey	9.67 ± 0.67	3.18 ± 0.17	5.44 ± 0.36*	0.23 ± 0.02	2.69 ± 0.02	2.46 ± 0.01^ns^
Onions	9.67 ± 0.88	3.04 ± 0.03	4.73 ± 0.06*	0.18 ± 0.02	3.08 ± 0.05	2.86 ± 0.06^ns^
Grapes	5.00 ± 0.00	3.13 ± 0.23	4.76 ± 0.07*	0.33 ± 0.02	3.10 ± 0.05	2.77 ± 0.01^ns^
Pickly pears C	4.33 ± 0.33	2.94 ± 0.16	5.00 ± 0.09*	0.48 ± 0.04	2.73 ± 0.04	2.61 ± 0.01^ns^
Pickly pears W	4.33 ± 0.33	2.94 ± 0.22	5.10 ± 0.30*	0.50 ± 0.04	2.72 ± 0.01	2.61 ± 0.03^ns^
Pickly pears A	3.33 ± 0.33	2.97 ± 0.18	4.96 ± 0.35*	0.60 ± 0.04	2.72 ± 0.01	2.61 ± 0.03^ns^
Tomatoes C	5.00 ± 0.00	2.53 ± 0.38	4.84 ± 0.31*	0.46 ± 0.02	3.02 ± 0.02	2.78 ± 0.02^ns^
Tomatoes W	5.00 ± 0.00	2.62 ± 0.67	5.04 ± 0.22*	0.48 ± 0.05	3.03 ± 0.06	2.75 ± 0.03^ns^
Tomatoes A	3.00 ± 0.00	2.97 ± 0.23	4.50 ± 0.42*	0.65 ± 0.12	2.99 ± 0.02	2.82 ± 0.05^ns^

*C, Control; W, Wood; A, Aerated. Statistical analysis has been performed using one-way ANOVA followed by Bonferroni post-hoc test for initial and final comparisons of acidity and pH; significance: ns, not significant,*

* *p < 0.0001*.

#### Vinegar Maturation by Accelerated Aging

The vinegar products produced from the six matrices with the conventional method were induced to accelerated aging by the addition of wood chips to the vinegar. The aerated and wood-fermented vinegar samples for prickly pear and tomato were not subjected to this accelerated aging in order to limit the variables and identify the cause of potential differences between the control (neat) vinegar and the vinegar subjected to accelerated aging with wood. For this study, standard French oak chips were employed together with cherry, as the latter imparts a sweet flavor to counterbalance the acidity of the vinegar. Changes in the vinegar samples after 15 days of contact time were validated by both organoleptic and instrumental analysis. Table 5 shows the main physicochemical characteristics of vinegar products produced from the six different substrates (grape, fig, tomato, honey, onion, and prickly pear) with different woods used for the maturation process (cherry, cherry/oak, oak, and control). For each substrate, all four treatments were compared statistically for any differences, and then in turn for each treatment. Finally, all substrates were compared for statistical differences. The tonality ratio represents the mixture of a color with white. Values ranged between 1.54 and 5.56 with no statistical significance. In principle, the absorbance values at 420 nm did not contribute to the variation between the matrices and treatments. On the other hand, parameters that depended on the 520 and 620, such as color intensity and anthocyanin content, were significantly affected. The color intensity represents the summation of the three prime colors at wavelengths 420, 520, and 620 nm. Although the color intensity values ranged between 0.52 and 18.29 A, the fig vinegar (14.07–18.29 A) and the prickly pear vinegar (5.86-5.94 A) products were significantly different from the other vinegar products (values < 2.53A) (*p* ≤ 0.01). These vinegar samples had intensely darker colors than the other vinegar samples. This was also reflected in the anthocyanin content. Values ranged between 1.32 and 67.40 mg/l. The most prominent vinegar samples were the ones obtained from figs (47.40–67.40 mg/l) and prickly pears (27.36–28.48 mg/l) when compared to the other samples (values < 12.37 mg/l) (*p* ≤ 0.01). Most of the wood-treated vinegar samples exhibited a low anthocyanin content when compared to the respective control, especially for the grape vinegar. This is consistent with Cerezo et al. (2010), concluding that the anthocyanins decrease with time, probably due to polymerization. The wood-treated samples had a consistently high content of phenols released by the wood with peak values resulting in the oak-treated samples (Table 5). The concentrations increased after 15 days of contact time with wood chips, except for the fig vinegar, whose phenolic content for the oak-treated vinegar was the lowest in value than the original mash. This can be attributed to the interaction between the bacterial cellulose and the phenolic content, as the latter is sequestrated by the cellulose fibers. This is concordant with Tang et al. (2003), who demonstrated the hydrophobic interaction between polyphenols and cellulose. Thus, the freshly produced vinegar has to be filtered to remove the cellulose fibers, and the microbial activity has to be deactivated through pasteurization to impede the polyphenol–cellulose fiber interaction. This step favors the retention of the maximum amount of phenolic content within the vinegar. However, fig vinegar (849–910 μg/ml) and prickly pear vinegar (1,346–1,794 μg/ml) exhibited significantly higher polyphenolic content than the other vinegar samples (values < 528 μg/ml) (*p* ≤ 0.01 and *p* ≤ 0.001). The correlation between matrices and treatments with respect to physicochemical content will be discussed later on. One of the major factors determining the quality of vinegar is the aging in wooden casks. The release of aromatic phenolic compounds from the wood, as it happens in the majority of the vinegar samples considered in this study, is higher in aged vinegar (Tesfaye et al., 2003, 2009; Callejón et al., 2010; Cerezo et al., 2010; Hidalgo et al., 2010). Moreover, the application of oak chips was found to accelerate the aging process, as reported by several authors (Tesfaye et al., 2003, 2009; Guerrero et al., 2011). Callejón et al. (2010) concluded that the preferred wood types for inducing aging in vinegar are oak and cherry for their rich, distinctive aromatic characteristics, particularly vanilla and wood correlated with oak, and red fruits and sweet with cherry (Mas, 2008). The presence of anthocyanins and other phenolic compounds in the finished product is directly related to the substrate type, maceration time, temperature, process method, sulfur dioxide presence, and alcohol content of wine among others, as confirmed by Dallas and Laureano (1994). Both these researchers and also Ivanova et al. (2010) concluded that the grape variety influences the level of the anthocyanins in the vinegar. The higher extractable anthocyanin portion is reflected in the chromatic characteristics of the final product. Thus, the level of anthocyanins in the finished product is not related to the high concentrations of anthocyanins in the berries but their extractability during the maceration process, as shown by Romero-Cascales et al. (2005). Most studies related to the anthocyanin content of vinegar are mostly based on the grape as substrate. There are very few studies showing this relationship with other substrates (Su and Silva, 2006; Wu et al., 2017; Chakraborty et al., 2018).

**Table 5:** Tonality ratio, color intensity, anthocyanin (mg/l), and polyphenolic (μg/ml) contents for the accelerated wood-aged vinegar samples.

**Vinegar**	**Treatment**	**Tonality ratio**	**Color intensity**	**Anthocyanins (mg/l)**	**Polyphenols GAE^**a**^ (μg/ml)**
Figs	Control	3.15 ± 0.05	18.29 ± 0.27*	67.40 ± 0.99*	901 ± 13.2*
	Cherry	3.64 ± 0.17	14.96 ± 0.69*	51.11 ± 2.35*	910 ± 41.8*
	Cherry/Oak	3.67 ± 0.07	14.07 ± 0.25*	47.40 ± 0.84*	898 ± 16.0*
	Oak	3.68 ± 0.10	14.80 ± 0.41*	49.71 ± 1.36*	849 ± 23.3*
Tomatoes	Control	2.41 ± 0.01	0.84 ± 0.00	3.42 ± 0.02	135 ± 0.6
	Cherry	2.54 ± 0.20	1.05 ± 0.08	4.18 ± 0.32	164 ± 12.6
	Cherry/Oak	5.56 ± 0.03	0.52 ± 0.00	1.32 ± 0.01	187 ± 0.9
	Oak	2.24 ± 0.02	1.26 ± 0.01	5.37 ± 0.05	204 ± 1.7
Honey	Control	2.08 ± 0.09	2.53 ± 0.11	11.82 ± 0.51	210 ± 9.1
	Cherry	2.61 ± 0.02	2.06 ± 0.02	8.72 ± 0.07	288 ± 2.3
	Cherry/Oak	2.37 ± 0.02	2.30 ± 0.02	10.05 ± 0.09	295 ± 2.5
	Oak	2.60 ± 0.17	1.77 ± 0.11	7.32 ± 0.46	297 ± 18.8
Onion	Control	1.93 ± 0.02	1.54 ± 0.01	7.24 ± 0.06	241 ± 2.0
	Cherry	2.46 ± 0.06	1.48 ± 0.03	6.11 ± 0.14	283 ± 6.4
	Cherry/Oak	2.43 ± 0.18	1.30 ± 0.10	5.76 ± 0.43	291 ± 21.5
	Oak	2.00 ± 0.04	1.74 ± 0.04	8.20 ± 0.18	301 ± 6.4
Prickly pear	Control	1.94 ± 0.59	5.86 ± 1.79*	27.97 ± 8.55*	1,346 ± 411.5**
	Cherry	1.86 ± 0.10	5.90 ± 0.31*	28.48 ± 1.48*	1,701 ± 88.1**
	Cherry/Oak	2.00 ± 0.23	5.94 ± 0.70*	27.95 ± 3.25*	1,704 ± 198.3**
	Oak	2.05 ± 0.11	5.87 ± 0.32*	27.36 ± 1.50*	1,794 ± 98.5**
Grapes	Control	1.54 ± 0.05	2.20 ± 0.08	12.37 ± 0.43	329 ± 11.4
	Cherry	1.93 ± 0.05	1.99 ± 0.05	10.18 ± 0.24	375 ± 8.7
	Cherry/Oak	2.16 ± 0.06	2.09 ± 0.06	9.97 ± 0.28	466 ± 12.9
	Oak	2.14 ± 0.17	2.39 ± 0.19	11.23 ± 0.89	528 ± 41.6

*GAE, Gallic acid equivalents. Statistical analysis has been performed using one-way ANOVA followed by Bonferroni post-hoc test for multiple mean comparisons; significance:*

* *p < 0.01*;

** *p < 0.001*.

**Figure 3:**
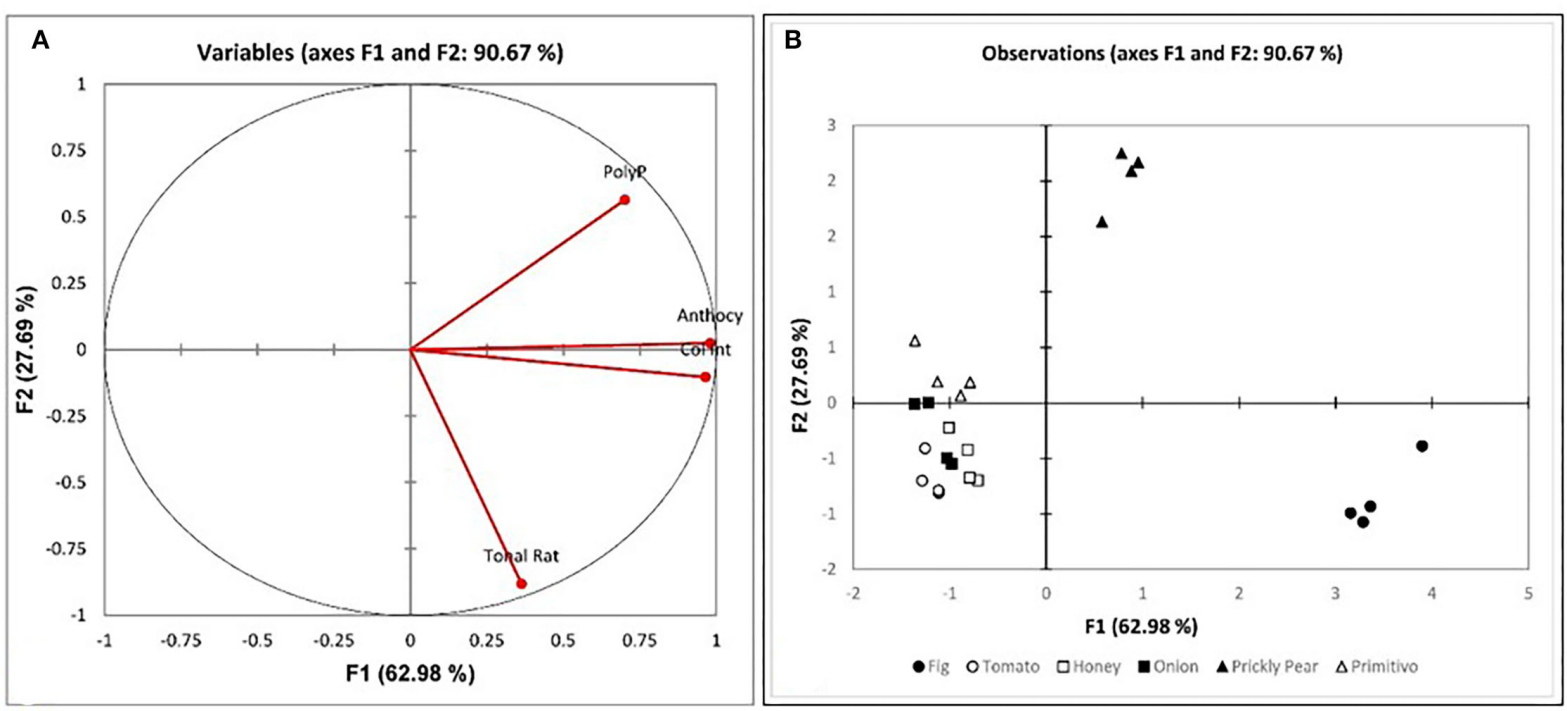
PCA analysis of the physicochemical parameters by matrix; **(A)** Variables plot with percentages of data variability for different substrate ferments (the accelerated wood-aged vinegar samples) of 90.67% is accounted for; **(B)** Score plot of the physicochemical variables and the chemical components of the various substrates at the end of the fermentation.

**Figure 4:**
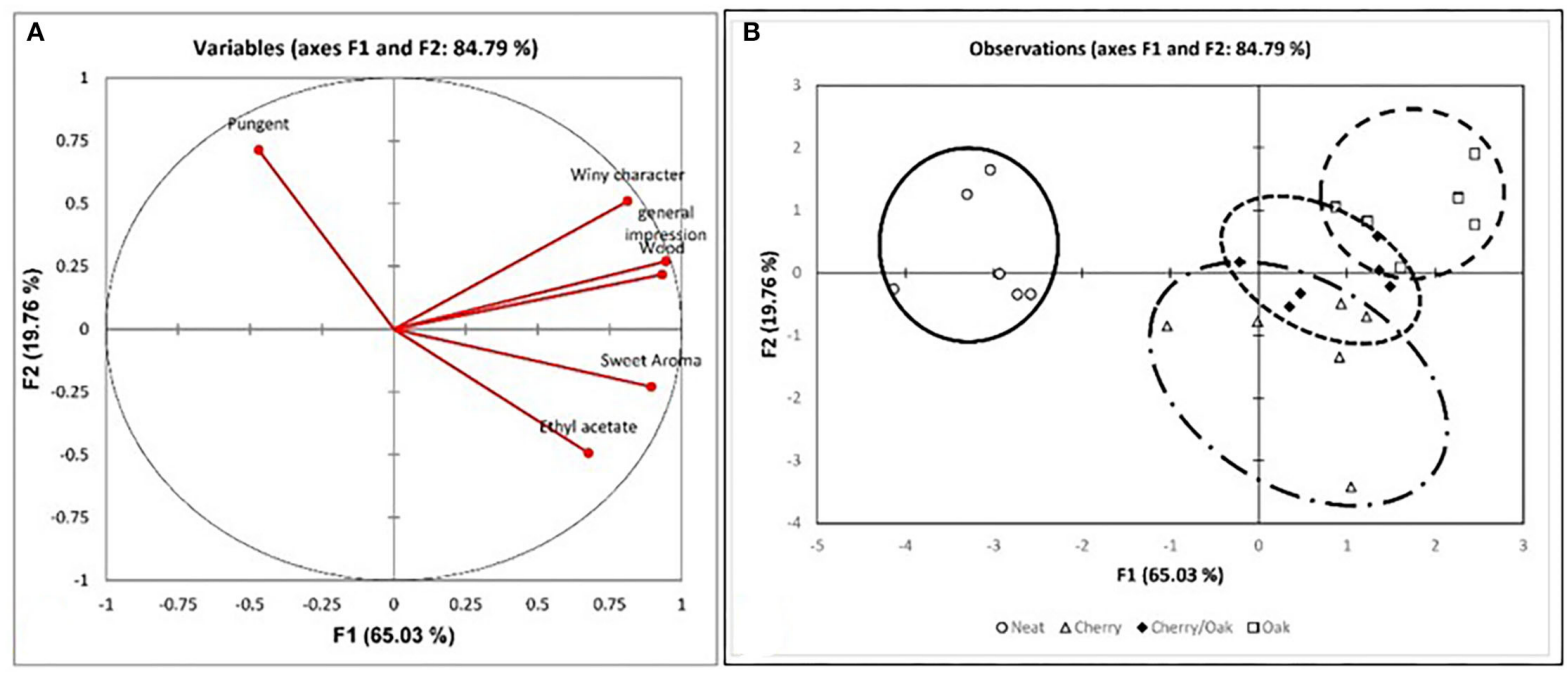
PCA analysis for the sensorial parameters for the six matrices by treatment, i.e., control and three wood treatments. **(A)** Variables plot for the sensorial parameters with a total variance of 84.79%; **(B)** observations plot of the four vinegar samples (control and the accelerated wood-aged vinegar samples) for the six matrices.

To further the investigation, correlation analysis was performed between phenolic compounds and the other parameters. Various cross-tab analyses were carried out to establish any correlation, both positive and negative, between the various datasets and establish any natural groups among the samples. The principal component analysis (PCA) was computed for the phenolic content, the anthocyanins, and the color intensity, as well as for the mean sensory evaluation of the vinegar sample. From the variables plot (Figure 3A), it can be assumed that there is a positive correlation between the color intensity and anthocyanin content (*r* = 0.989), as these all are clustered in one direction. For the principal components, F1 is represented by the physical properties of the non-flavonoids, including the phenolic acids and the flavonoids, i.e., the anthocyanins expressed as the color intensity and tonality. The F2 is represented by the chemical properties of the polyphenols. The clustering in the observations plot (Figure 3B) shows that while tomatoes, honey, grapes, and onions exhibited low values for anthocyanins (<12.37 mg/l), prickly pears and figs exhibited much higher values (>27.36 mg/l), and the honey correlated more strongly with the physicochemical tests, the acidity and pH. On the other hand, the prickly pear exhibited superior total polyphenolic content (>1,346.19%, w/w) when compared to all other matrices (<910.28% w/w). In addition to the physicochemical analyses, the organoleptic evaluation of the vinegar samples was performed. The results showed the distinction between the controls and the wood-treated vinegar samples, with the wood-treated vinegar being of a commercial grade, whereas all controls except for the fig were categorized as a domestic grade. The Pearson correlation coefficient (r) reveals a correlation between the wood and both the general impression and sweet aroma in Figure 4A (*r* = 0.933 and *r* = 0.723, respectively). This loading plot clearly demonstrated that the pungency is inversely correlated with the sweet aroma (*r* = −0.502). The reason behind this is that the wood-treated vinegar samples had the pungency masked by the mellower attributes the wood imparted into the vinegar, with the sweet aroma contributed by the wood, and the general impression giving the highest contrast. This is in accordance with Tesfaye et al.'s (2010) findings where the pungency correlated negatively with the sweet aroma sensation. The score plot in Figure 4B shows the position of the mean score obtained by the seven panelists for the neat and the accelerated wood-aged vinegar samples. The controls are clustered opposite the oak for the F1 axis. Apart from one cherry wood matured sample, all the other wood-treated vinegar samples give a higher score than the control or neat vinegar samples. Another distinctive clustering is observed in the neat vinegar samples and the cherry matured vinegar samples. However, the oak and cherry/oak treatments clearly exhibited a distinctive group. This confirms that the sweet cherry aroma and oak flavor released by the wood into the vinegar highly contrasts the pungent aroma and flavor of the neat vinegar samples.

## Conclusion

This study aimed at describing the characteristics of vinegar produced from the available raw materials from local Maltese agricultural and food processing and retail facilities. The whole production process was considered step by step to identify the most efficient way forward. Autochthonous strains of AAB were isolated and used together as acetous fermentation starters. Finally, the qualities imparted by the wood maturation process were also investigated to complete the whole vinegar production process. Although the study did not rely on the appropriate selection of bacterial strains based on their functional and technological properties, it has demonstrated that all the substrates can be successfully employed in the production of vinegar, obtaining a product that is appreciated by consumers, complementing the vast list of condiments as part of the rich traditional Mediterranean culinary culture. The substrates can be both surpluses and/or by-products of a farm, food processing plants, food retail outlets, and food stores. Exploiting these materials and converting waste into a resource renders the system more sustainable not only for industry but also for the individual farmers, the cottage industry, and even a domestic application whereby the leftovers are utilized, reducing the load on the municipal refuse collection. Vinegar biotechnology is the result of environmental resources, culture, tradition, and science. The vast array of substrates from which vinegar can be produced leads the way for product diversification, based on the complex tasting habits of today‘s consumers, and it is the driving force for new product development with different organoleptic and sensorial characteristics. Although chaptalization can be somehow an added cost (when necessary), the use of different local raw materials, especially by-products of agro-industry, brand the local quality vinegar production as “zero kilometer” and sustainable, and the local vinegar production can be categorized as a low-carbon footprint niche industry with exponential growth potential.

## Data Availability Statement

The datasets presented in this study can be found in online repositories. The names of the repository/repositories and accession number(s) can be found at: NCBI GenBank–OM984645-OM984649.

## Author Contributions

JM and EA conceived the study, performed the chemical analysis, and prepared the statistical analysis. JM, FG, and NB carried out the microbiological analysis and sequence elaboration. JM, FG, NB, DD, and EA contributed to the writing of the paper. FG and DD critically revised the final manuscript. All authors contributed to the article and approved the submitted version.

## Conflict of Interest

The authors declare that the research was conducted in the absence of any commercial or financial relationships that could be construed as a potential conflict of interest.

## Publisher's Note

All claims expressed in this article are solely those of the authors and do not necessarily represent those of their affiliated organizations, or those of the publisher, the editors and the reviewers. Any product that may be evaluated in this article, or claim that may be made by its manufacturer, is not guaranteed or endorsed by the publisher.

## Supplementary Material

Supplementary Figure 1 Restriction profiles of isolated AAB strains with *Alu*I enzyme.

Supplementary Figure 2 Restriction profiles of isolated AAB strains with *Hae*III enzyme.

Supplementary Figure 3 Acidity and pH measures during three cycles of grape **(A)**, fig **(B)**, honey **(C)**, and onion **(D)** acetous fermentations.

Supplementary Figure 4 Acidity and pH measures during three cycles of prickly pear (PP) acetous fermentations: control **(A)**, wood **(B)**, and aerated **(C)**.

Supplementary Figure 5 Acidity and pH measures during three cycles of tomato (T) acetous fermentation: control **(A)**, wood **(B)**, and aerated **(C)**.

Supplementary Table 1 Clustering of isolated strains based on ARDRA fingerprinting and restriction analysis with *Alu*I and *Hae*III.

Supplementary Table 2 Pearson correlation values for the comparison between the pH and acidity at the beginning and end of the acetous fermentation.
