# Supplementary figures and images for: Selection of Acetic Acid Bacterial Strains and Vinegar Production From Local Maltese Food Sources
Source: Front Microbiol. 2022 Jul 19;13:897825. doi: 10.3389/fmicb.2022.897825 (PMC9343879; doi:10.3389/fmicb.2022.897825)

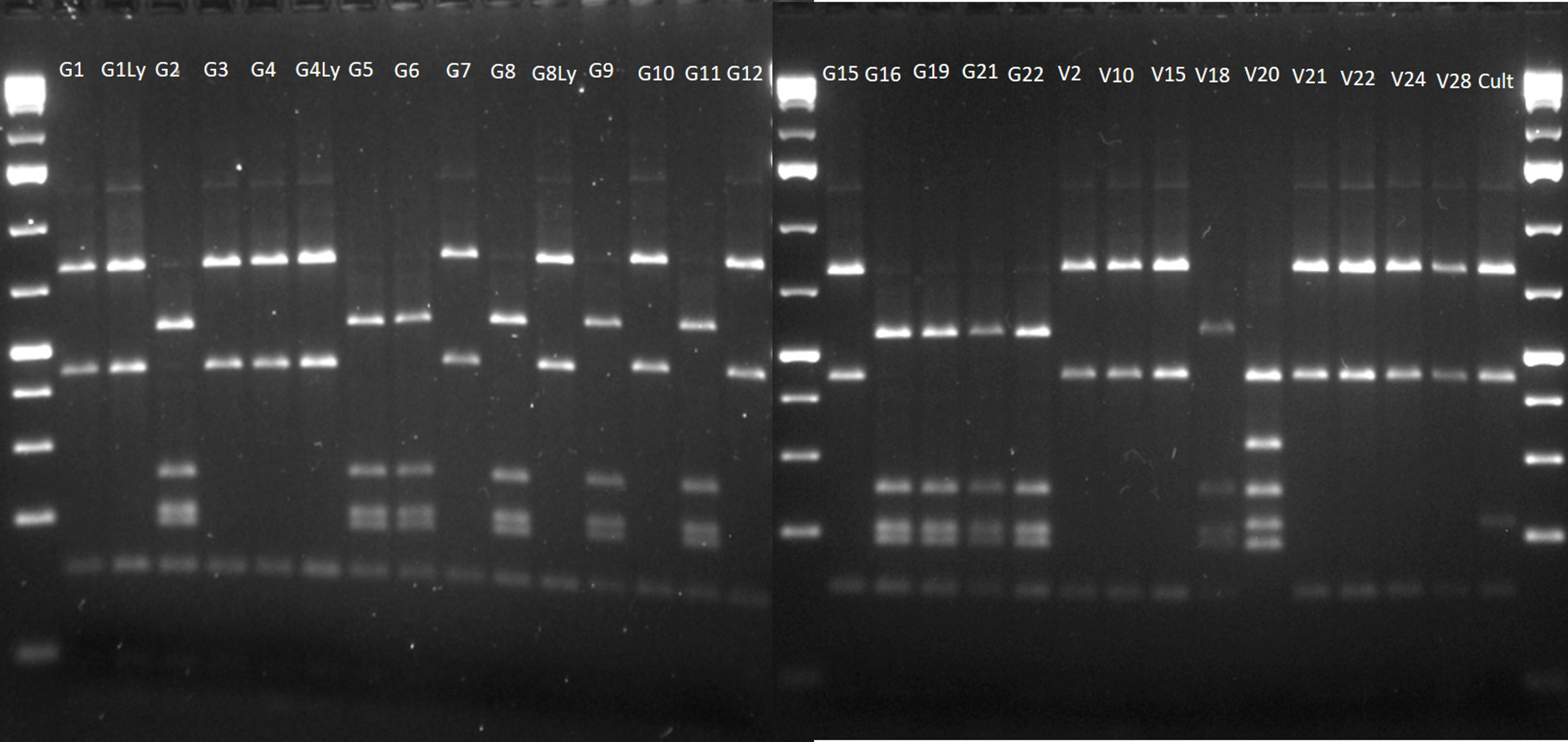

Supplement: Supplementary Figure 1 — Restriction profiles of isolated AAB strains with AluI enzyme. [file Image_1.JPEG]

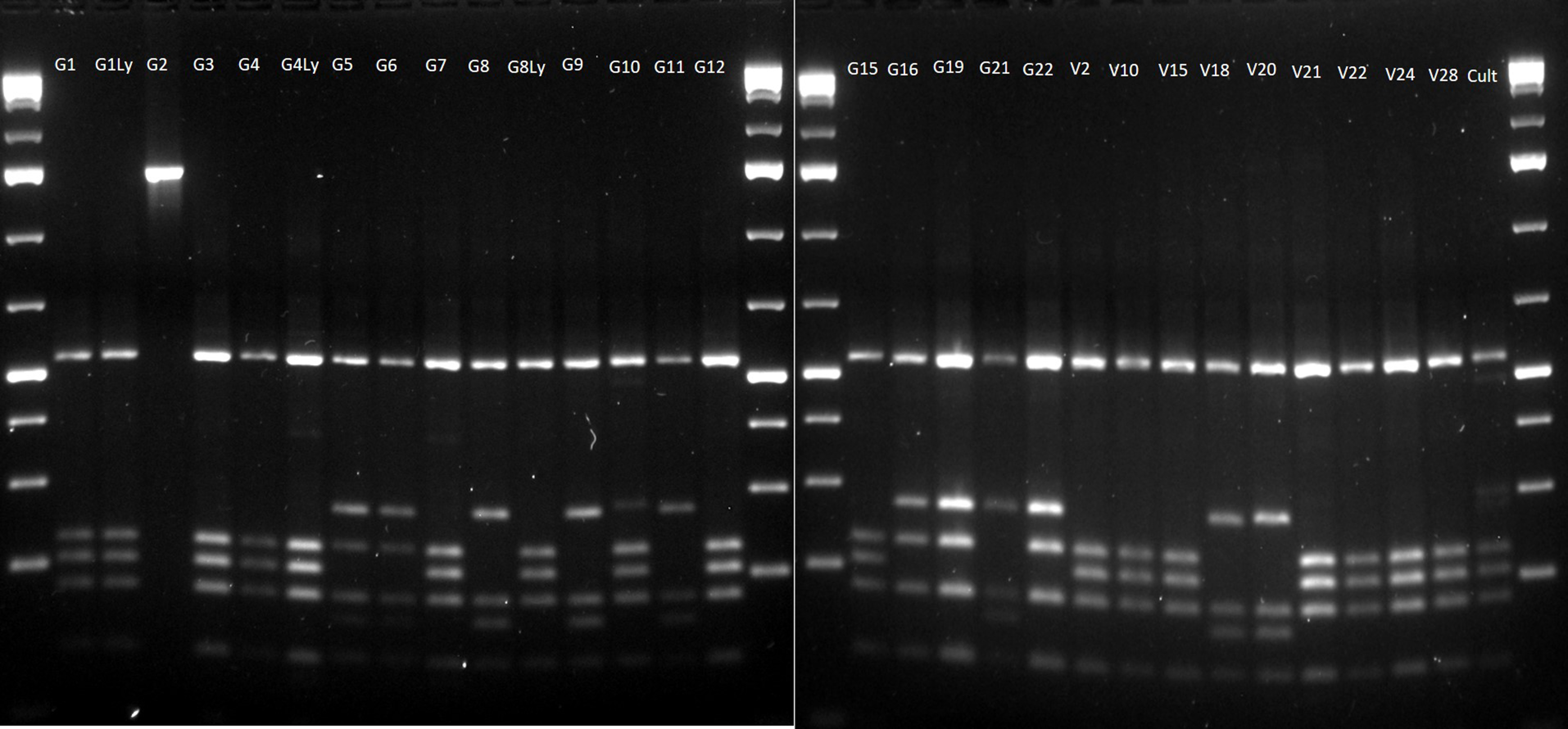

Supplement: Supplementary Figure 2 — Restriction profiles of isolated AAB strains with HaeIII enzyme. [file Image_2.JPEG]

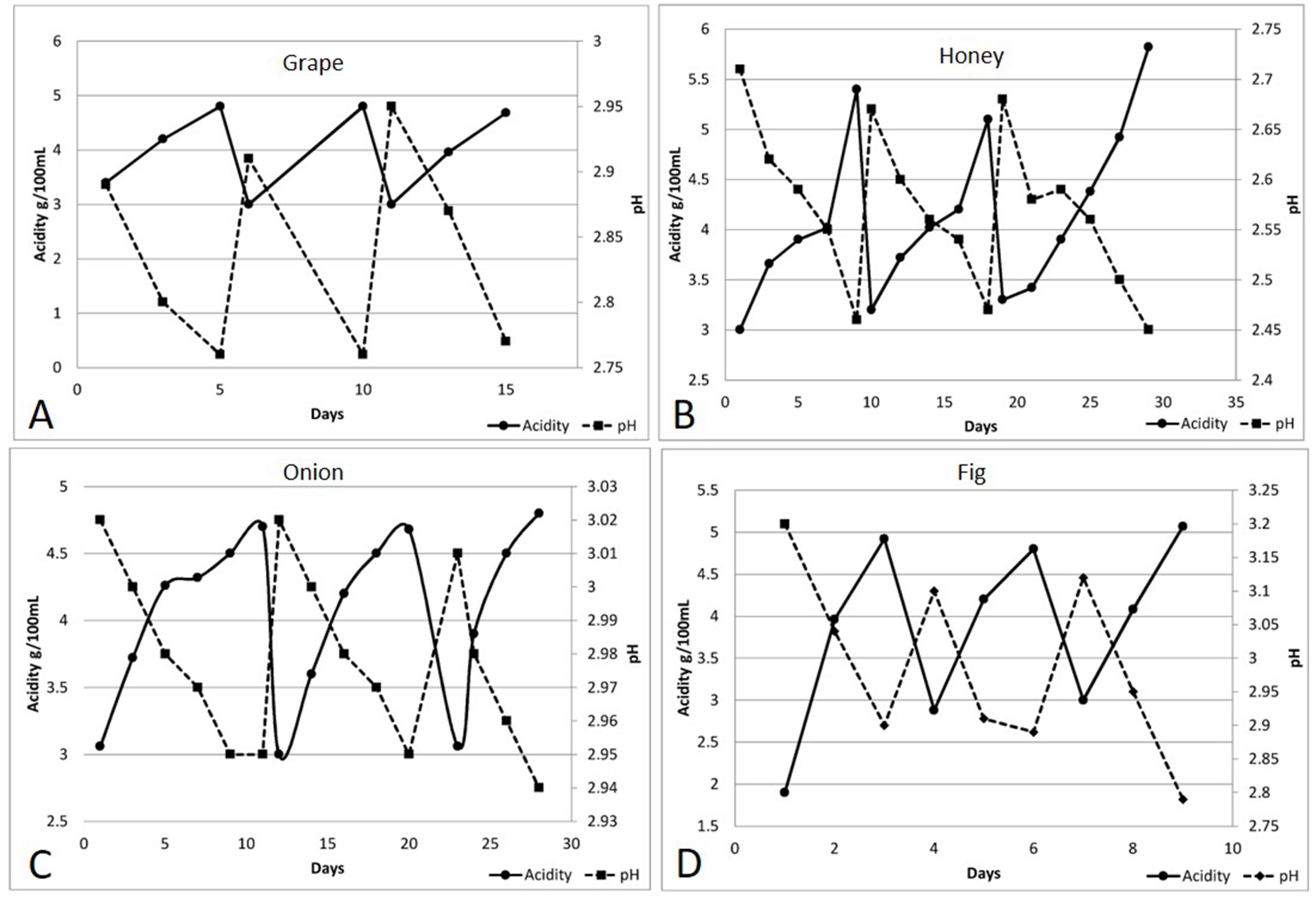

Supplement: Supplementary Figure 3 — Acidity and pH measures during three cycles of grape (A), fig (B), honey (C), and onion (D) acetous fermentations. [file Image_3.JPEG]

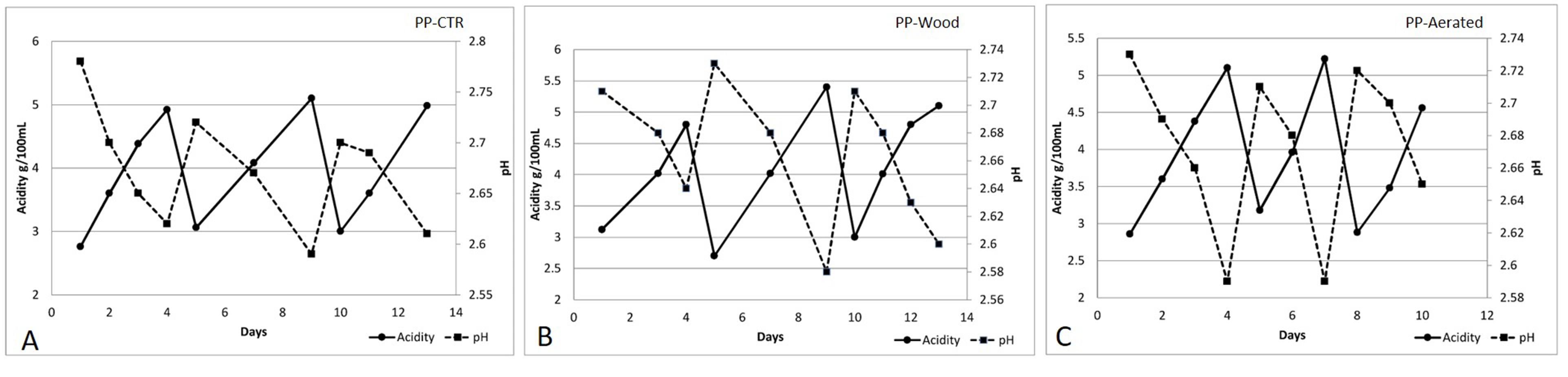

Supplement: Supplementary Figure 4 — Acidity and pH measures during three cycles of prickly pear (PP) acetous fermentations: control (A), wood (B), and aerated (C). [file Image_4.JPEG]

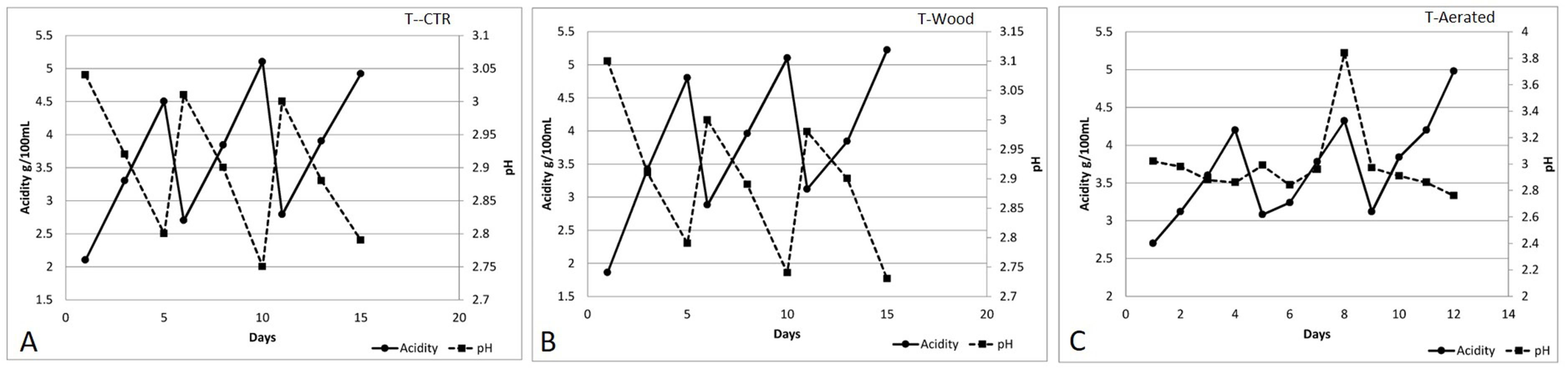

Supplement: Supplementary Figure 5 — Acidity and pH measures during three cycles of tomato (T) acetous fermentation: control (A), wood (B), and aerated (C). [file Image_5.JPEG]
