# Supplementary material for: Selection of Acetic Acid Bacterial Strains and Vinegar Production From Local Maltese Food Sources
Source: Front Microbiol. 2022 Jul 19;13:897825. doi: 10.3389/fmicb.2022.897825 (PMC9343879; doi:10.3389/fmicb.2022.897825)
Supplement: Supplementary Table 1 — Clustering of isolated strains based on ARDRA fingerprinting and restriction analysis with AluI and HaeIII. [file Table_1.DOCX]

**Table S1**. Clustering of isolated strains based on ARDRA fingerprinting and restriction analysis with *Alu*I and *Hae*III

| *Alu*I |  | *Hae*III |  |
| --- | --- | --- | --- |
| Cluster 1 | G1, G11Ly, G3, G4, G4Ly,G7, G8Ly, G10, G12, G15, V2, V10, V15, V2, V10, V15, V21, V22; V24, V28 | Cluster 1 | G1, G11Ly, G3, G4, G4Ly,G7, G8Ly, G12, G15, V2, V10, V15, V2, V10, V15, V21, V22; V24, V28 |
| Cluster 2 | G2, G5, G6, G8, G9, G11, G16, G19, G21, G22, V18 | Cluster 2 | G10 |
| Cluster 3 | V20 | Cluster 3 | G2, G8, G9, G11, G21, V18, V20 |
|  |  | Cluster 4 | G5, G6, G16, G19, G22 |
