# Supplementary material for: Selection of Acetic Acid Bacterial Strains and Vinegar Production From Local Maltese Food Sources
Source: Front Microbiol. 2022 Jul 19;13:897825. doi: 10.3389/fmicb.2022.897825 (PMC9343879; doi:10.3389/fmicb.2022.897825)
Supplement: Supplementary Table 2 — Pearson correlation values for the comparison between the pH and acidity at the beginning and end of the acetous fermentation. [file Table_2.docx]

**Table S2.** Pearson correlation values for the comparison between the pH and acidity at the beginning and end of the acetous fermentation.

|  | | pH/acidity  start | pH/acidity  finish |
| --- | --- | --- | --- |
| Prickly Pears | Control | -0.908 | -1.000 |
|  | Wood | -0.961 | 1.000 |
|  | Aerated | -0.893 | -0.985 |
| Tomatoes | Control | -0.993 | -0.847 |
|  | Wood | -1.000 | -0.992 |
|  | Aerated | -0.949 | -0.999 |
